# Supplementary material for: Invariant death
Source: F1000Res. 2016 Aug 25;5:2076. [Version 1] doi: 10.12688/f1000research.9456.1 (PMC5063037; doi:10.12688/f1000research.9456.1)
Supplement: Peer review history

# Peer review: Referee response for version 1

**DOI:** 10.5256/f1000research.10185.r16246

**Published:** 2016-10-07

**Authors:** Ophélie Ronce

**Affiliations:** 1 French National Center for Scientific Research, University of Montpellier, Montpellier, France

This paper by S. A. Frank builds on his recent work on general common patterns of statistical distributions to comment on some invariance rules when examining the distribution of mortality data. In particular, the present paper shows that there always exists an adequate transformation of time (or age), such as, when measured on this scale, the shape of the survival curve is unaffected by any shift on this scale. Interestingly, Frank here also shows that on this scaling, the survival curve has necessarily a Gompertz form. On this scaling, the acceleration of mortality with (transformed) time is constant.

 This is indeed interesting to realize that any mortality distribution would have these properties. So not much could be inferred about the mechanisms of mortality and aging just because one transformation of time would lead to the distribution of mortality satisfying those properties. The remaining question is whether we can infer something from the fact that one transformation of time rather than another produces this invariance.

 As an illustration, the present paper by S. A Frank then comments on a recent study by Stroustrup *et al.* (2016, hereafter S2016), which showed that various genetic or environmental interventions affected mortality patterns in nematodes populations by modifying the time scale of mortality, leaving the shape of survival curves unchanged. More precisely, a log transformation of time then satisfies the invariance properties mentioned above. Interestingly, a few interventions in their data set do not exhibit invariance after a log-transformation of time. In the supplementary material of the S2016, the authors have examined a diversity of mechanistic models of mortality and checked under which perturbations of the parameters of these models one would observe the specific invariance pattern seen in their data. In some of these models, the specific invariance pattern emerges only when all sub-processes affecting mortality are affected to the same extent by the intervention (e.g. competing risk models), or when all parameters in the models are affected to a similar extent by the intervention (diffusion models). In some other models (e.g. network models), the specific invariance can emerge when only part of the processes are affected by the intervention. These conclusions appear therefore more subtle than the claim made in the main text of S2016 that “each intervention must alter to the same extent throughout adult life all physiological determinants of the risk of death”. It is furthermore not very clear to me what is meant exactly by “all physiological determinants of the risk of death”. Can we say that the drift and variance terms in a diffusion model for vitality correspond to different physiological determinants of the risk of death? Again we could imagine different mechanistic sub-models that would create different functional relationships between the drift and variance parameters.

 Frank uses S2016 in two ways: first, as an illustration of a particular type of invariance exhibited by real data, second, as a warning against the risk of over-interpretation of such invariances. In particular, Frank reacts to the claim cited above, which is indeed confusing. As S2016 did in their supplementary material, Frank exhibits a mechanistic model of mortality (but yet a different one from those considered S2016), which shows the same properties of invariance when only part of the processes of mortality are affected by the intervention. Both the theoretical exercise of Frank and those of S2016 show that there are many different mechanistic models that can exhibit the same specific invariance and thus raise together strong doubts about what we can infer from invariances in mortality patterns. The set of models examined by S2016 is certainly not exhaustive despite its diversity, and claims based upon this set (already more complex than the simple cited argument suggests) cannot really be generalized to all models of mortality.

 I therefore agree with the general message of caution of the present paper by S.A. Frank. It however remains frustrating that the general framework that he proposes does not really help to get a better general grasp at what features of a model would produce one type of invariance rather than another, and for instance help generating hypotheses that would explain why some interventions did fail to produce the same type of invariance in S2016. This what S2016 attempted in their supplementary material.

 In particular, I would be interested in a clearer illustration of the claim made by Frank that “consideration of the alternative processes with the same observable invariances leads to testable predictions about the underlying causal processes”.

 Interestingly, the model put forward by Frank is shift-invariant on a scale, which is not a simple log-transformation of time (see equation 17 and that following on the right column of page 6). While early in the process, the deformation of time to achieve invariance would resemble that expected under a log-scaling, this is not true later. I therefore failed to understand exactly why this model would actually fit the data presented by S2016. More explanation would be necessary here.

 The whole argument page 7 about interventions affecting part of the processes when slow and fast processes determine mortality makes intuitive sense but would be more convincing if illustrated (as was done for instance with examples in the supplementary material of S2016). I found these arguments about slow and fast processes to be quite disconnected actually from the general arguments about invariance presented before.

 To conclude, I found the contribution of Frank novel and interesting, but still a bit frustrating about how this perspective could help us extract more information from patterns of mortality. The message of caution is an important one. The criticism of S2016 is justified by the over-simplistic claim included in their abstract and main text, but I am a bit concerned that it may misrepresent what these authors have achieved. This claim was actually motivated, not directly by the examination of invariance in the data, but by the comparison of several models to data in a spirit similar to what Frank proposes here. That several models could exhibit the same invariance rule for different reasons was clearly shown already in S2016 (supplementary material). The common properties of models exhibiting that specific invariance rule however appear even less clear than suggested by the initial exploration of S2016 and it could be good that the present paper communicates more precisely about this point.

I have read this submission. I believe that I have an appropriate level of expertise to confirm that it is of an acceptable scientific standard.

# Peer review: Referee response for version 1

**DOI:** 10.5256/f1000research.10185.r15879

**Published:** 2016-09-14

**Authors:** Sean Nee

**Affiliations:** 1 The Pennsylvania State University, Pennsylvania, PA, USA

Survival analysis is a field with a long history, mainly associated with the name David Cox, and is of interest far outside of biology, e.g. the reliability analysis of machines. One element of it concerns making inferences about underlying mechanisms from observed processes. The historical development of the subject seems to have ended in a cul-de-sac which we see in Stroustrup's worm paper, discussed here, although clearly explored in an earlier Ricklefs paper, discussed by no-one. Frank offers an entirely new approach with a different starting point. It seems sensible to discuss the Frank paper at the junction where the new collides with the old, which is the Stroustrup worm paper, published in *Nature *magazine this year.

 The heart of the matter, from my perspective, is this. The old development of the subject ended up at this point -

1. Survival data is typically well described by *both* Weibull and Gompertz distributions, which are described in terms of the 'hazard' function derived from them, describing your future chance of surviving beyond a particular time, given you have survived to that time so far.
2. If these hazards change for some reason (e.g. temperature for Stroustrup), we can model the effect of these changes in one of two ways, dreamed up by Cox because they 'work' for statistical analysis. One of these ways (AFT) is a time scaling of the hazard, in which everything happens in 'dog years' for example. The other (PH), as noted by Cox, is very hard to give a biological interpretation to in terms of underlying mechanisms, although it is very useful for prediction, evaluating drug efficacy etc.

Now, the Weibull distribution can be proved analytically to allow interpretation under both these ways of changing circumstance, one of which is, itself, intrinsically hard to interpret. So if the data are Weibull, which fits the Stroustrup data, you cannot really say anything about the underlying mechanism. Unless you get very inspired and allow "imagination" (Ricklefs) and "preferences" (Stroustrup Supp Info) to assist you. This is not necessarily a bad thing, of course, but it sounds like an admission that you have gone as far as you can by the traditional route. I note that the Supp. Info Figure 1.1, which is central to the narrative flow, uses a Weibull distribution, which has a log-log hazard and the Weibull plays a large role in their statistics/simulations etc. The Gompertz has a measly log hazard, which rather screws up the simple scaling story.

 Frank now approaches the whole subject in a way which is new, thought-provoking, challenging and very welcome, starting from a rethinking of the basic meanings of statistical distributions. It is far to soon to have an opinion on the likely success of this approach in leading us out of the cul-de-sac. But I certainly have the opinion it should be published.

 The Ricklefs paper deserves some acknowledgement and will also flag this topic for a larger audience of evolutionary ecologists, as it concerns the evolution of longevity, species variation in longevity etc. etc.

 Ricklefs RE, Scheuerlein A. 2002. Biological implications of the Weibull and Gompertz models of aging. J Gerontol A Biol Sci Med Sci. 2002 Feb;57(2):B69-76.

 doi: 10.1093/gerona/57.2.B69

I have read this submission. I believe that I have an appropriate level of expertise to confirm that it is of an acceptable scientific standard.

## References

[1] RicklefsRScheuerleinA: Biological Implications of the Weibull and Gompertz Models of Aging. *The Journals of Gerontology Series A: Biological Sciences and Medical Sciences*.2002;57(2) : 10.1093/gerona/57.2.B69 B69-B76 10.1093/gerona/57.2.B69 11818426

[2] StroustrupNAnthonyWENashZMGowdaVGomezALópez-MoyadoIFApfeldJFontanaW: The temporal scaling of Caenorhabditis elegans ageing. *Nature*.2016;530(7588) : 10.1038/nature16550 103-7 10.1038/nature16550 26814965PMC4828198
